# Essential Business Visits and Social Vulnerability during New York City’s Initial COVID-19 Outbreak

**DOI:** 10.3390/epidemiologia3040039

**Published:** 2022-11-10

**Authors:** Debra F. Laefer, Delphine Protopapas

**Affiliations:** 1 Center for Urban Science & Progress, Tandon School of Engineering, New York University, Brooklyn, NY 11201, USA; 2 Department of Civil and Urban Engineering, Tandon School of Engineering, New York University, Brooklyn, NY 11201, USA; 3 Weston Research Scholars Program, Brooklyn Technical High School, Brooklyn, NY 11217, USA

**Keywords:** social vulnerability, healthcare facilities, COVID-19, New York City, essential workers, points of interest

## Abstract

New York City (NYC) was deeply impacted by COVID-19 in spring 2020, with thousands of new cases daily. However, the pandemic’s effects were not evenly distributed across the city, and the specific contributors have not yet been systematically considered. To help investigate that topic, this study analyzed the interaction of people with neighborhood businesses and other points of interest (POIs) in parts of three NYC neighborhoods in the spring of 2020 during the peak of the first COVID-19 wave through anonymized cellphone data and direct the observation of 1313 individuals leaving healthcare facilities. This study considered social vulnerability index (SVI) levels, population density, and POI visit behaviors from both cellphone data and firsthand observations of behavior around select NYC health facilities in different boroughs as various proxies. By considering equivalent businesses or groups of businesses by neighborhood, POI visits better aligned with COVID-19 infection levels than SVI. If tracking POI visit levels proves a reliable direct or relative proxy for disease transmission when checked against larger datasets, this method could be critical in both predictions of future outbreaks and the setting of customer density limits.

## 1. Introduction

On 8 April 2020, New York City (NYC) reported a 7-day average of 5482 new COVID-19 cases and 1583 new hospitalizations [1] representing the peak of the pandemic in the city during the first 20 months. This high outbreak level occurred despite a state-level Pause Order that came into effect 17 days earlier, which ordered non-essential businesses to suspend in-person operation [2]. While many stayed at home, most healthcare, transit, food-sector, and other essential workers could not, and subsequently became ill. Although there has been documentation of the disproportionate levels of COVID-19 infections among frontline workers [3] and those from socially disadvantaged communities [4], the exact details of community transmission are less well-known [5]. To this end, this study investigated visits to essential businesses during spring 2020 as a proxy to understand disproportionate community transmission of COVID-19 in specific NYC neighborhoods.

“Social vulnerability refers to a community’s capacity to prepare for and respond to the stress of hazardous events ranging from natural disasters, such as tornadoes or disease outbreaks, to human-caused threats,” [6] according to the Center for Disease Control (CDC). The CDC’s numeric social vulnerability index (SVI) is composed of 15 factors from census data that are categorized into four themes: (T1) socioeconomic status, (T2) household composition and disability, (T3) minority status and language, and (T4) housing type and transportation [6]. Each census tract across the United States is given an SVI rating based on these factors, with 0 as the least vulnerable and 1 as the most vulnerable. In NYC, there are 2099 census tracts, with an average SVI of 0.625 and standard deviation of 0.265 [7].

The CDC’s prediction that an already vulnerable community is more likely to be severely impacted by a natural disaster or public health crisis has been widely demonstrated across decades of natural disasters [8]. Since spring 2020, this has also been seen to some extent with COVID-19 infection rate disparities. In various cities across the United States, collectively, COVID-19 infection and death rates are higher in more socially vulnerable areas [4]. NYC, taken as a whole, is no different (Table 1). A Chi-squared goodness-of-fit test was performed to determine whether the proportion of COVID-19 case ranges was equal between 10 groups of SVI levels. The significance threshold was set at 0.05. The proportions differed by SVI level, X^2^(9, *N* = 2097) = 417.38, *p* < 0.001.

The first wave of COVID-19 rapidly spread through NYC, despite unprecedented control efforts. Specifically, effective 22 March 2020, New York State’s PAUSE shuttered “all non-essential businesses statewide” [2]. Only essential health care operations, transit and utility infrastructure, essential manufacturing and services, and retail related to food provision, pharmacies, household goods, and hardware were permitted to continue to operate [11]. Most healthcare facilities and many of the other “essential” businesses remained open during this time. Their uninterrupted operation required the majority of their staff to come to work, thereby forcing many to commute via public transportation at a time where the asymptomatic transfer of COVID-19 was not widely recognized, and mask wearing was not yet recommended. Consequently, essential workers were often infected at work and while commuting, which led them to carrying COVID-19 into their homes and their neighborhoods [12]. Coincident with the continued, in-person operation of essential businesses, many of NYC’s wealthier residents left the city. In some affluent neighborhoods, this was as much as 40% [13]. This mass exodus did not occur in socially vulnerable neighborhoods. Some of Manhattan’s busiest stations had as little as 20% of their previous ridership during spring 2020, whereas outer boroughs with more essential workers retained as much as 50% of their pre-pandemic ridership [14].

Furthermore, researchers have noted that people in socioeconomically vulnerable neighborhoods may have been financially constrained from buying online or stockpiling food [15,16], which may have translated into more frequent trips to obtain food or longer durations spent in stores. Additionally, the existence of food deserts in many socially vulnerable neighborhoods with the higher numbers of small convenience stores and fast-food stores and fewer grocery stores translated into a higher number of visitors per store [17].

Poverty was not the only factor for higher COVID-19 infection rates. Socioeconomically vulnerable neighborhoods have a higher percentage of racial and ethnic minority residents, who are more likely to be employed in “essential” jobs [18]. Essential workers such as those in emergency services, transportation, and grocery stores generally could not work from home, thereby potentially exposing themselves to COVID-19 on a near daily basis. 

**Figure 1:**
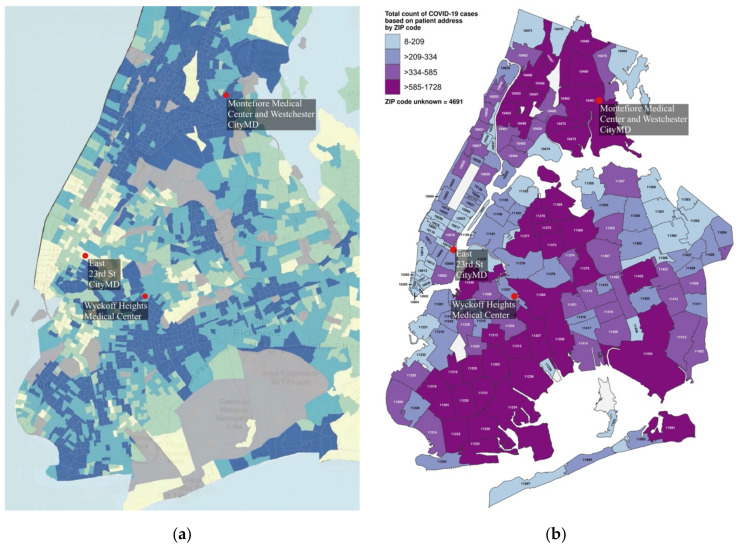
SVI and COVID-19 infection rates on 8 April 2020 in The Bronx, Brooklyn, Manhattan, and Queens (red dots indicate study locations): (**a**) SVI ratings (blue, more vulnerable; yellow, less vulnerable) [7]; (**b**) total counts of COVID-19 by case ranges by zip code [10].

Although COVID-19 infection rates in NYC track with social vulnerability levels (Figure 1), there is a paucity of research documenting the detailed behaviors that have led to this. Without further study, the problem of community transmission will remain poorly understood. To begin to bridge this research gap, this study considered visit levels at select groups of businesses in communities with varying social vulnerability levels.

Multiple studies have established a link between the higher prevalence of COVID-19 infections and social vulnerability; however, few have attempted to more granularly explore the exact transmission mechanisms. This paper builds on the well-established concept of food deserts in socially vulnerable areas [19,20] as a driver for higher visit densities to food-related businesses, thereby transforming these locations into potential transmission hotspots. Direct linkage between the number of daily visitors to individual businesses would affirm this as a viable direction for larger-scale studies, because such information could better inform the development of public health policies by imposing occupancy restrictions. This study explored this concept through two mechanisms (anonymized cell phone data and direct field observations) for select neighborhoods in New York City during the first wave of COVID-19 in spring 2020. This pandemic was unprecedented in the modern age; therefore, various proxies for transmission spread are considered

This paper first presents an overview of the research to date (Section 1). Next, Section 2 introduces the data used in the study, the methods of analysis, and the three study locations. The results are presented in Section 3. This is followed in Section 4 with a discussion of those results with respect to the current state of knowledge and the limitations within the study itself. Finally, Section 5 presents the conclusions that can be drawn from this study, the actions that could be taken to mitigate the spread of infection based on these conclusions, and further research that should be carried out in this area.

## 2. Materials and Methods

This section provides the rationale for the sites selected, the data sets employed, and the specific analysis approaches adopted. This study considered in-person patronage for portions of three socioeconomically distinct NYC neighborhoods as a proxy for exposure to COVID-19. Nearby points of interest (POIs) were considered; a POI is a specific, physical location that someone may find interesting (e.g., restaurants, retail stores, and grocery stores) [21]. The research herein considered POIs that were the same or similar across the three sites, as well as in-person patronage levels of POIs for a handful of businesses in the immediate vicinity of three selected NYC healthcare facilities. 

**Figure 2:**
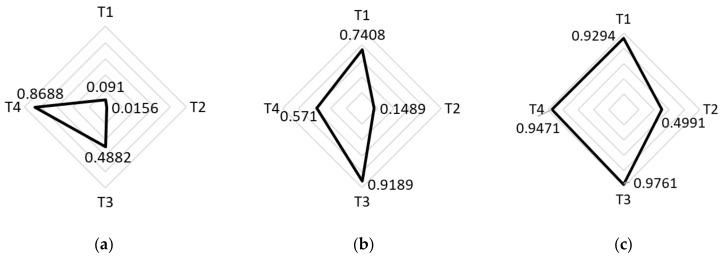
SVI by theme where T1 is socioeconomic status, T2 is household composition and disability, T3 is minority status and language, and T4 is housing type and transportation [7]: (**a**) Manhattan, Tract 64, SVI = 0.2083; (**b**) Brooklyn, Tract 443, SVI = 0.6854; (**c**) Bronx, Tract 200, SVI = 0.9610. # = number.

Three locales were selected from different boroughs to be emblematic of disparities across NYC as represented by SVI ratings (Figure 1a) and COVID-19 infection rates (Figure 1b), with a Manhattan location with a low SVI and low COVID-19 levels, a Bronx location with a high SVI and high COVID-19 levels, and a Brooklyn location with a mid-level SVI and mid-level COVID-19 infection rates (Table 2). The NYC Department of Health counted COVID-19 cases by zip code and not census tract; therefore, zip code level data were used for infection rate information (Figure 1b), whereas the more precise census tract level data were used for assessing social vulnerability. An overview is presented in Figure 2.

The analysis involved four parts. The first compared 2020 versus 2019 in-person visits at a McDonald’s operating in each locale and located closest to an operational healthcare facility (Figure 1) to establish some parity between locations. The selection of a McDonald’s was based on the concept of the “Big Mac Index” being a useful mechanism of comparison between socioeconomically different countries due to the status of the global chain’s signature product as a “perfect universal commodity” [23]. This logic was extended to compare visit behaviors, as opposed to specific buying patterns or comparative costs. In this case, anonymized cell phone location data provided for POIs were taken from the “Monthly Places Patterns” datasets from the SafeGraph Consortium (sometimes known as Placekey) [24]. Visits are based on pings collected voluntarily from specific cellphone applications for approximately 20% of all cell phones in New York State [25]. The data are organized by location record, affiliated POI addresses, and quantity of daily visits per POI. A Jupyter notebook with the Pandas data science library [26,27] was used to retrieve the data of interest. Weekly averages in 2019 were compared with those in 2020, beginning with the week 15–21 March (immediately preceding the PAUSE order) and for the next 10 weeks through the week of 24–30 May 2020. The significance level was set at 0.05.

The second analysis also used anonymized cellphone data to consider operating businesses within 150 m of the main entrance of the healthcare facilities using the street address column as a filter. POIs with zero daily visits from April to May 2020 were assumed to be non-operational and excluded from the analysis. Percentages of the previous year’s visits were calculated for three different weeks: a normal week at the beginning of February, the week preceding the official NYC PAUSE order, and a week in April around the height of cases in the city; the first COVID-19 case in NYC was reported on 1 March 2020 [28]. Next, from within these, a set of “standard businesses” was picked to examine comparable businesses. For this, the fast-food chain Subway sandwich shop was used, because chains are likely to have similarly sized locations, usage, and operating hours. The other two types of businesses were a local (non-chain) café and a non-chain deli/grocery. SafeGraph data for the weeks of interest mentioned above were collected and compared for the third analysis.

**Figure 3:**
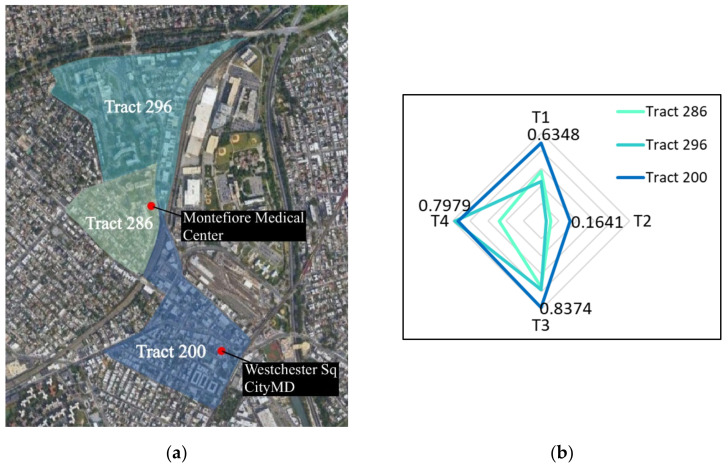
Community characteristics around Montefiore Medical Center: (**a**) satellite image overlain by tract boundaries and their population for tri-partite population areas surrounding Montefiore Medical Center; (**b**) visualized weighted SVI for areas in 3a, where T1 is socioeconomic status, T2 is household composition and disability, T3 is minority status and language, and T4 is housing type and transportation.

The fourth analysis considered in-person observations that were collected as part of a National Science Foundation (NSF) study of egress behaviors outside healthcare facilities at the onset of the pandemic (Figure 3).

That study, entitled DETER: Developing Epidemiology mechanisms in Three-dimensions to Enhance Response, documented numerous personal choices including personal protective equipment (PPE) usage, touch behaviors, destinations, and transportation choices of individuals leaving 19 healthcare facilities across 16 NYC zip codes during the period 23 March to 17 May 2020 [1]. The observers followed randomly selected individuals for up to 20 min or 1 mile or until visual contact with the subject was lost. DETER data were not available for The Bronx urgent care facility; therefore, the closest DETER location was selected. This was the Montefiore Hospital, which was only 0.56 miles away and in the same zip code but at the edge of three census tracts (Figure 3a). The tabulated data of the individual SVI themes are shown in Table 3 with a weighted average based on population shown in Figure 3b; the hospital had insufficient surrounding commercial facilities to use that location for all analyses. From the DETER data, the percentage of people visiting specific POIs was monitored for the weeks following the initiation of the PAUSE order.

## 3. Results

**Figure 4:**
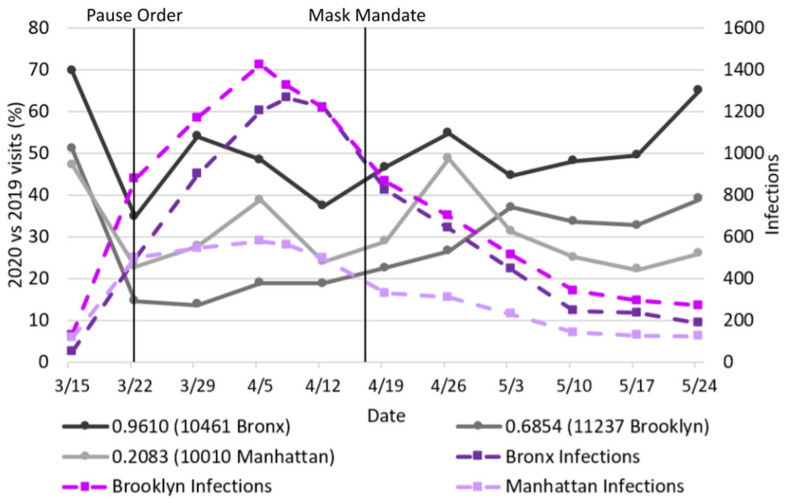
Seven-day average of the percentage of the previous year’s daily visits (15 March to 30 May 2019 vs. 2020) at 3 NYC McDonald’s, alongside the total 7-day rolling average infection case counts for Brooklyn, Bronx, and Manhattan [29].

Figure 4 shows the total percentage change over the previous year differing by location, over the 11-week period for the trio of selected McDonald’s. Week 2 (which included the onset of PAUSE order) had the fewest visits across all sites, and for two of the three locales there was a notable dip in Week 5, which coincided with the peak city-wide infection level; the reported daily infection levels are provided for contextualization.

The McDonald’s in The Bronx (SVI = 0.9610) consistently had a higher percentage of visits with respect to the previous year than the other two locations; never decreasing below 35%, and by the end of the 11 weeks reaching 65% (50% on average for the 9 weeks following the PAUSE order). In contrast, the McDonald’s in the least socially vulnerable neighborhood (Manhattan; SVI = 0.2083) bottomed out at 22%, was highly variable, and ended the observation period at a visit level around half that in The Bronx at only 26%, with an overall average across the 9 weeks following the PAUSE order of 30%. The Manhattan numbers may also be impacted by the absence or decrease in workers commuting to that borough.

Although the McDonald’s in Brooklyn (SVI = 0.6854) had an average of only 27% in the 9 weeks following the PAUSE order, the trend was quite different. Visit levels started out having the lowest percentage at 15%, but increased significantly over time and reached 39% in the final week. Over the 11 weeks, Manhattan had 31%, Brooklyn 27%, and The Bronx 48% of visits of that of the previous year. Based on SVI levels, the Brooklyn numbers are surprisingly low, although this may be indicative of extra caution being taken in that neighborhood due to the local hospital having the unfortunate distinction of being the first in NYC to receive temporary morgue wagons, which were stationed immediately adjacent to the hospital’s staff entrance starting 28 March 2020 [30]. Although infection rates in that neighborhood were not in the highest category, that healthcare center is the primary hospital for many of the neighborhoods directly east, which were more severely infected (Figure 1b).

**Figure 5:**
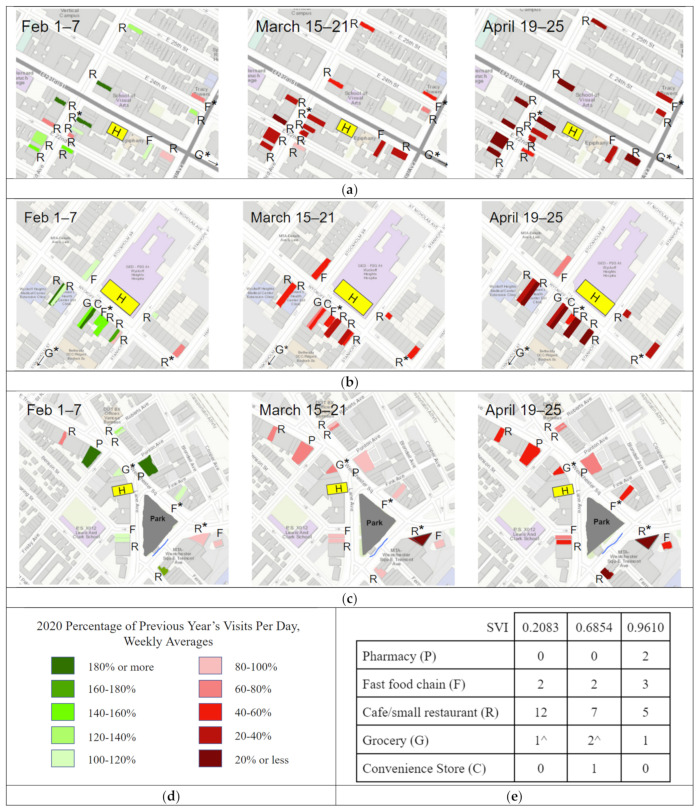
The 2020 percentage of the 2019 year’s visits per day for a set of businesses near 3 healthcare facilities (weekly averages). R, cafe or small, independent restaurant; G, deli or grocery; F, national fast-food chain; H, healthcare facility; * indicates that a business’s data was used in the following graph, ^ indicates that a business was not pictured on the map, but it was nearby and included in the total set of POIs listed in the sub-captions. (**a**) 23rd St CityMD: Manhattan, 10,010; 15 POIs; SVI = 0.2083; (**b**) Wyckoff Heights Medical Center: Brooklyn, 11,237; 12 POIs; SVI = 0.6854; (**c**) Westchester Square CityMD: The Bronx, 10,461; 11 POIs; unweighted SVI = 0.9610; (**d**) legend for sub-figures (**a**–**c**); (**e**) facilities per study area.

For the businesses near open healthcare facilities (one per neighborhood), the SafeGraph POI visit level data were visualized as a percentage change between 2019 (a normal year) and 2020 (Figure 5). There was an overall decrease in POI visits among both smaller businesses and major chains in all three locales for the weeks after February (Figure 5); however, there were disparities between the Manhattan (Figure 5a), Brooklyn (Figure 5b) and The Bronx (Figure 5c) sites in terms of how quickly people stopped visiting nearby businesses and the loss of patronage by mid-April. In February, the maps look nearly the same. The second week was prior to the PAUSE order, but after the in-person closures of NYC universities [31] and the announcement of the suspension of in-person public school instruction starting 16 March [32]. In that week, the businesses in the least vulnerable neighborhood had already lost the majority of their visitors, as shown by the dark red. This was in contrast to the much slower visit losses in less affluent areas. At the Manhattan site (SVI = 0.2083), by mid-March, 64% of the examined business experienced at least a 60% reduction in visits, and by mid-April 57% had lost at least 80%. These losses were noticeably less in Brooklyn. In mid-March, all of the Brooklyn site businesses experienced at least a 40% decline, but only 20% saw a 60% reduction and none saw a reduction greater than 80%.

Fisher’s exact test was used to determine significance between the SVI of the sites and the number of businesses with more than a 50% decrease in visits from February to March. When comparing the Manhattan and The Bronx locations, there was a statistically significant association between borough and the number of businesses with more than a 50% decrease in visits from February to March (*p* = 0.04).

**Figure 6:**
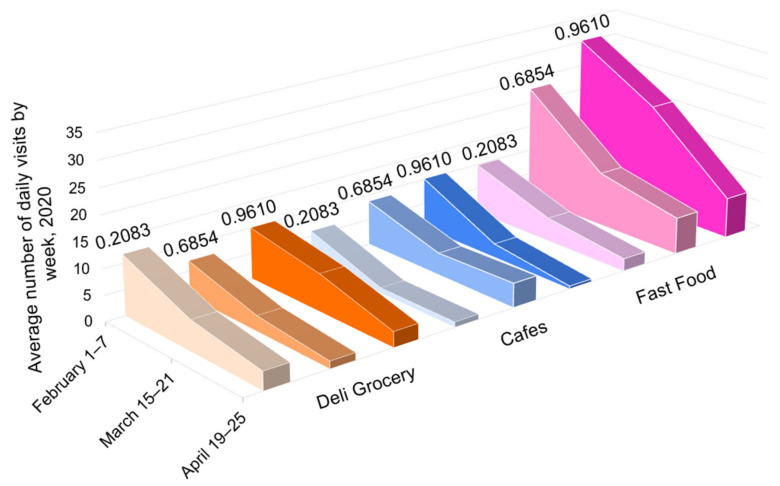
Daily visits per location for food-related POIs for 23rd St CityMD (SVI 0.2083), Wyckoff Heights Medical Center (SVI 0.6854), and Westchester Square CityMD (unweighted SVI 0.9610), based on select cell phone data.

Within each sub-image in Figure 5, three businesses are starred. These were considered as comparable businesses across locales and are featured in Figure 6: small independent grocery stores in orange, non-chain cafes or restaurants that offered take out in blue, and the fast-food chain Subway in pink. The darker shading corresponds to both the greater vulnerability and higher infection rate for the area. Table 4 shows the 2020 visit levels as a percentage of those for the same week in 2019.

In the week of 15–21 March, the decrease in visit levels in businesses in the least vulnerable area exhibited much greater losses (−58% in Manhattan vs. −35% in Brooklyn and −40% in The Bronx). By mid-April, in the week following peak infection levels, all locations were experiencing a loss of 62–77% of their customer visits (Table 4). Critically, the percentages only tell part of the story, as shown in Figure 6, where the total visits for these three businesses for the weeks in March and April were 260, 436, and 598 in order of neighborhood social vulnerability, with much of the disparity being in the fast-food establishments. In the week in April, across the three business types, this translated to visits per POI of 5.13, 7.75, and 10.35 in order of SVI. Arguably, the numbers can be considered as a proxy for infection rate, as shown in Table 2.

**Table 4:** Percentage change comparing 2020 and 2019 visits per location for food-related POIs for 23rd St CityMD (SVI 0.2083), Wyckoff Heights Medical Center (SVI 0.6854), and Westchester Square CityMD (SVI 0.9610), based on select cell phone data.

	Grocery (G)			Restaurant/Cafe (R)			Fast-Food (F)		
SVI	0.2083	0.6854	0.9610	0.2083	0.6854	0.9610	0.2083	0.6854	0.9610
1–7 Feb.	+52%	+81%	+28%	+124%	−35%	−7%	−16%	+58%	+23%
15–21 Mar.	−40%	−1%	−26%	−78%	−57%	−80%	−56%	−48%	−14%
19–25 Apr.	−72%	−63%	−70%	−87%	−65%	−95%	−71%	−57%	−63%

**Figure 7:**
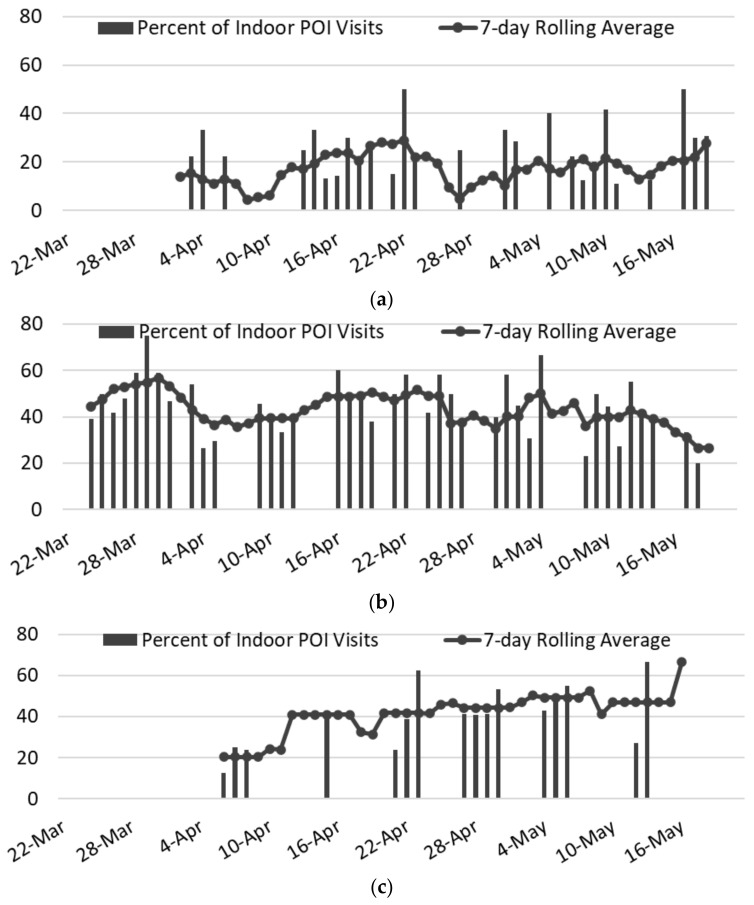
Percentages of people visiting an indoor POI location after leaving each of the three selected health facilities, during the DETER observation period of 22 March to 17 May 2020. (**a**) East 23rd St CityMD, SVI = 0.2083 (33 POIs 4.4–28.9%); (**b**) Wyckoff Heights Medical Center, SVI = 0.6854 (28 POIs 26.7–56.8%); (**c**) Montefiore Medical Center, weighted SVI = 0.7991 (4 POIs 20.4–66.7%).

The final analysis considered the destination choice of individuals leaving healthcare facilities; note the weighted average SVI for the location in The Bronx (see Table 3). Figure 7 charts the rate of indoor POI visits; the 7-day rolling average is a better indicator than the individual daily records, which were collected opportunistically based on staff availability. The strongest observable trend was in the overall visit levels within each neighborhood, which reflected trends seen in the SafeGraph data. Specifically, in the least vulnerable neighborhood, only 4.4–28.9% of those leaving the healthcare facility entered an indoor POI, despite there being 33 indoor POIs open. At the mid-vulnerability location, which had 28 operational indoor POIs, the visit rate was 26.7–56.8%. In the slightly more socially vulnerable area, the visit rates were similar, but there were only 4 indoor POIs

As shown in Table 5, although the total number of subjects observed in the least vulnerable area was nearly identical to that of the most vulnerable area (335 subjects versus 347), not only did the percentage of individuals visiting an indoor POI differ (19.1% for Manhattan vs. 41.8% for The Bronx), but the number of people per POI exhibited a nearly 18-fold difference (1.9 vs. 36.3). The POI visit level in the two locales with SVIs slightly above NYC’s average appeared highly similar (43.4% (Brooklyn) vs. 41.8% (The Bronx)), but the number of people per indoor POI in Brooklyn was one-third of what it was in The Bronx, even though the total number of individuals was much greater (631 in Brooklyn vs. 347 in The Bronx), because of the paucity of POIs in the more socially vulnerable location, with only four Bronx locations versus 28 in Brooklyn. When looking at the COVID-19 cases, the visits per POI were arguably better predictors of the relative level of COVID-19 cases than SVI or population density. Fisher’s exact test was used to determine significance between SVI level and the number of days with a higher rate of indoor POI visits than the average across all dates and locations. Significance was found (*p* < 0.001) when the Manhattan site was compared with the more socially vulnerable locations in Brooklyn and The Bronx, but not when Brooklyn was compared with The Bronx.

## 4. Discussion

Many studies have associated socioeconomic vulnerability with higher rates of COVID-19 and greater mobility in the early weeks of the pandemic (often using POI visits as a proxy). For example, in a study of 38,000 POIs in Los Angeles, census block groups with higher SVIs (typically between 0.5 and 0.75) had both greater mobility and higher COVID-19 infection rates, although the authors did not provide specific quantitative cut-offs in their “low”, “medium”, and “high” classifications [33]. In a study of POIs across all of NYC, in the early weeks of the 2020 PAUSE order, higher SVI levels and greater mobility levels were initially associated with larger community COVID-19 positivity rates, but those correlations rapidly declined by the end of the PAUSE order’s sixth week [34]. In an aggregate study of POIs across the United States’ ten largest metropolitan areas, higher infection rates were present among more socioeconomically disadvantaged areas, which was attributed to differences in mobility levels [35]. Additionally, those authors simulated the potential positive impact that capping POI occupancy to pre-pandemic levels would have in reducing the spread of COVID-19 [35]; however, there was no consideration of the actual POI visit levels compared with those of the previous year or the real 2020 visit levels and their potential correlations with actual neighborhood infection rates. Although POI visits do not capture the entirety of a community’s population, they have been used as a reliable proxy for mobility (as shown above), and Safegraph estimates that their data collection represents 20% of all cellphone usage in the State of New York.

**Figure 8:**
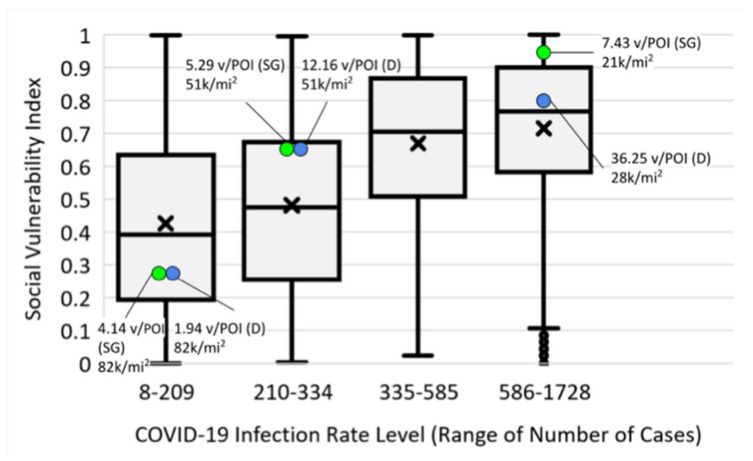
SVI versus COVID-19 infection rate levels on 8 April 2020 (x = mean of each distribution for all locations in Figure 1b; D, DETER data and SG, SafeGraph data for 2–8 April 2020 for the 3 McDonald’s detailed in Figure 4). Green dots show visit levels from SG data, and blue dots represent first-hand observations in the studied communities (as shown in Figure 2).

By analyzing NYC’s communities at a census tract level, each SVI (Figure 1a) was assigned a COVID-19 infection rate from the more aggregated zip code level (Figure 1b) for 8 April 2020 (the peak of NYC infections). Even though this date is only 17 days into the PAUSE order, the use of SVI as a definitive predictor for COVID-19 infection levels is not particularly robust, as shown by the high level of overlap of SVIs in each of the COVID-19 levels (Figure 8). Although the argument can be made that there is an inherent bias on COVID-19 reporting based on testing availability being greater in less socially vulnerable areas, in reality, testing was typically not available in this period, except at hospitals. Thus, further factors are clearly needed, and as shown in Figure 8, local, urban population density cannot be used, at least not using numbers reported through census-type mechanisms, as opposed to checking the location of phones typically resident in a neighborhood or through some dynamic means such as the level of residential trash collection.

By concentrating on the number of individuals per POI (Table 5), a more nuanced predictor at a hyper-local level may be possible. Clearly, the next step would be to perform such analysis at scale, such as by considering visits per similar POIs or groups of POIs (e.g., all fast-food restaurants assuming that indoor activity could be distinguished from drive-through). Similarly, if such correlations prove valuable, then hourly POI visit rates should be determined to determine whether that and the square meterage of a POI could be used to establish a customer density as a better predictor and a potentially direct input to capping occupancy. The higher POI visit levels may mirror underlying “food desert” situations where local residents do not have easy access to grocery stores, and therefore may visit POIs such as fast-food restaurants more regularly [17]. The pandemic may also have brought about or significantly exacerbated local “food deserts”, further influencing the POI visit behaviors in the initial weeks of COVID-19.

As the United States struggles with ongoing waves of COVID-19 and considers mitigation efforts against future pandemics, knowledge of these differing behaviors and why they occur could help to reduce the potential harm to specific communities and be a potential indicator of where to pre-assign resources for communities that are likely to be most impacted. The indication that high visit levels at individual POIs and groups of specific POIs correlates well with COVID-19 infection levels considered in this study highlights the importance of having more widespread access to essential goods and services, as a way to reduce POI visit concentration levels. Furthermore, essential workers (such as those working at healthcare facilities) could be provided with resources to help them avoid interacting with nearby businesses when traveling to and from work and during breaks over the course of their workdays.

## 5. Conclusions

This study considered indoor POI visits at a hyper-local level in select NYC communities of varying SVI, COVID-19 infection, and population density levels. This was performed specifically nearby operational healthcare facilities through the analysis of footfall data collected from approximately 20% of all New York State cell phones and analysis of the direct observation of 1313 individuals leaving healthcare facilities in the period February–May 2020. By focusing on comparable, indoor POIs in the form of name-brand fast-food restaurants and small groups of other POIs in the immediate vicinity of healthcare facilities, a more equalized view of behaviors at the onset of COVID-19 in NYC could be considered. 

Many of the observations mirrored more generic, large-scale studies such as higher mobility generally linked to higher SVI levels; for example, areas of high SVI levels had indoor POI visit levels that were slower to drop and never decreased as starkly as less socially vulnerable communities. However, in the case of NYC, commonly touted factors of influence for COVID-19 infection levels were shown not to be definitive predictors (e.g., SVI level) or not at all influential (e.g., pre-COVID-19 population density levels). In contrast, this study demonstrated that the density of POI visits was able to explain vastly different COVID-19 infection levels in communities that were demographically similar. This was shown with (1) named fast-food chains, (2) a small set of standard stores, and (3) a larger set of businesses around select health facilities. This was true even when hyper-local events (e.g., the deployment of temporary morgue wagons outside of hospitals) appeared to influence local choices about visiting indoor POIs.

A potential rationale for these results is that more socioeconomically vulnerable neighborhoods are also commonly known to be “food deserts” [19], where residents do not have access to large grocery stores offering a variety of fairly priced, fresh, and healthy food. In low-income neighborhoods of NYC, the stores that offer food are usually smaller bodegas [20]. These neighborhoods have fewer and smaller grocery businesses; therefore, residents are more concentrated near these businesses, which the research herein found to be correlated with higher COVID-19 infection rates.

This study is limited by the uncertainty of whether the visitors to these retail establishments are neighborhood residents, workers, or passersby. However, robust proxies for transmission spread risk have not yet been established rigorously, and exploring different methods of transmission prediction can provide useful information for future pandemic mitigation efforts. In this study, multiple proxies were analyzed in an effort to contribute to the dialogue of establishing appropriate proxies for urban COVID-19 transmission. 

The results indicate that occupancy levels of essential businesses could play an important role. Airborne transmission is the primary method of COVID-19 spread; thus, socially distancing was recommended, but not constraints on occupancy limits or levels. Potentially, a factor could be developed as a percentage of already established allowable occupancy under current fire codes [36]. Arguably, such a metric could be simply and rapidly implemented into public health guidelines. However, validating such an approach would require knowledge of the quantity of publicly accessible floor space within each POI, which is not a readily available piece of information. Alternatively, the current allowable occupancy levels may be viable proxies for the square meterage, although this would not take into consideration ceiling height or air exchange equipment, both of which could contribute to the mitigation of air-borne disease transmission. 

## Figures and Tables

**Table 1:** New York City SVI information [9] compiled from Figure 1 below.

Neighborhoods by SVI	Number of Census Tracts	Average Lower Bound of COVID-19 Cases (per 100,000 Residents)	Average Upper Bound of COVID-19 Cases (per 100,000 Residents)
SVI < 0.625	925	311	757
SVI > 0.625	1174	441	1155

**Table 2:** Comparison of demographics and COVID-19 rates in selected locations.

Location’s Borough	Zip Code (# of Census Tracts)	Population Density (per Square Mile)	Median Annual Income [22]	COVID-19 Case Range per 100,000 People [10]	SVI [7] (Specific Tract #)	COVID-19 Cases [10]
Manhattan	10010 (9)	81,487	USD 140,869	8–209	0.2083 (64)	Low
Brooklyn	11237 (14)	50,927	USD 74,990	209–334	0.6854 (443)	Med. Low
Bronx	10461 (19)	21,045	USD 38,199	585–1728	0.9610 (200)	High

**Table 3:** Tabulated weighted SVI for three census tracts around Montefiore Medical Center.

Tract Number	Population	SVI	Population Density (People/mi^2^)
286	1085	0.3769	14,662.5
296	1575	0.6058	9148.1
200	4711	0.9610	37,853.8
Average (Weighted by Population)	---	0.7991	---

**Table 5:** Observations of people visiting an indoor POI location after leaving each of the three selected health facilities, during the DETER observation period of 22 March to 17 May 2020.

SVI	Subjects	Rate (Subjects/Hour)	Indoor POIs	Subjects that Visited POI (%)	Visits/POI Possible	Visits/POI Observed
0.2083	335	2.3	33	19.10	10.15	1.94
0.6854	631	5.9	28	43.42	22.54	12.16
0.7991 *	347	5	4	41.79	86.75	36.25

* weighted.

## Data Availability

The author-affiliated data are available at Laefer, D.F., Kirchner, T., Jiang, H. Egress Behavior from Select NYC COVID-19 Exposed Health Facilities March–May 2020. https://doi.org/10.17609/smpm-3c34 and http://hdl.handle.net/2451/60075 (accessed on 17 August 2022).

## References

[1] New York City (NYC) Gov (2022). COVID-19: Data Trends and Totals. NYC Health COVID-19 data. https://www1.nyc.gov/site/doh/covid/covid-19-data-totals.page#zip.

[2] New York State Department of Health (2020). New York State on Pause. NY Department of Health. https://coronavirus.health.ny.gov/new-york-state-pause.

[3] The Lancet (2020). The plight of essential workers during the COVID-19 pandemic. Lancet.

[4] Khazanchi R., Beiter E.R., Gondi S. (2020). County-Level Association of Social Vulnerability with COVID-19 Cases and Deaths in the USA. J. Gen. Intern. Med..

[5] Andersen L.M., Harden S.R., Sugg M.M., Runkle J.D., Lundquist T.E. (2021). Analyzing the spatial determinants of local Covid-19 transmission in the United States. Sci. Total Environ..

[6] Centers for Disease Control and Prevention (CDC) (2020). At a Glance: CDC Social Vulnerability Index (SVI). Centers for Disease Control and Prevention. https://www.atsdr.cdc.gov/placeandhealth/svi/index.html.

[7] CDC (2018). Social Vulnerability Index (SVI) Interactive Map. Centers for Disease Control and Prevention. Svi.cdc.gov/map.html.

[8] Palaiologou P., Ager A.A., Nielsen-Pincus M., Evers C.R., Day M.A. (2019). Social vulnerability to large wildfires in the western USA. Landsc. Urban Plan.

[9] Governor’s Press Office (2020). Governor Cuomo Issues Guidance on Essential Services Under The ‘New York State on PAUSE’ Executive Order. NY Governor. https://www.governor.ny.gov/news/governor-cuomo-issues-guidance-essential-services-under-new-york-state-pause-executive-order.

[10] Schuchat A. (2020). Public Health Response to the Initiation and Spread of Pandemic COVID-19 in the United States, February 24–April 21, 2020. MMWR Morb. Mortal Wkly Rep..

[11] Quealy K. (2020). The Richest Neighborhoods Emptied out Most as Coronavirus Hit New York City. New York Times. https://www.nytimes.com/interactive/2020/05/15/upshot/who-left-new-york-coronavirus.html.

[12] Goldbaum C. (2020). Crowded Subways? Yes, in Neighborhoods Where People Have to Go to Work. New York Times. https://www.nytimes.com/2020/12/02/nyregion/subway-ridership-mta.html.

[13] CDC (2021). Centers for Disease Control and Prevention/Agency for Toxic Substances and Disease Registry/Geospatial Research, Analysis, and Services Program. CDC/ATSDR Social Vulnerability Index 2018 Database New York. https://www.atsdr.cdc.gov/placeandhealth/svi/data_documentation_download.html.

[14] NYC Gov (2020). Total count of COVID-19 cases based on patient address by ZIP code, April 8 2020. NYC Health COVID-19 Data Archive. https://www1.nyc.gov/assets/doh/downloads/pdf/imm/covid-19-cases-by-zip-04082020-1.pdf.

[15] Yoshizaki H.T.Y., de Brito I., Hino C.M., Aguiar L.L., Pinheiro M.C.R. (2020). Relationship between Panic Buying and per Capita Income during COVID-19. Sustainability.

[16] Nicola M., Alsafi Z., Sohrabi C. (2020). The socio-economic implications of the coronavirus pandemic (COVID-19): A review. Int. J. Surg..

[17] Hilmers A., Hilmers D.C., Dave J. (2012). Neighborhood disparities in access to healthy foods and their effects on environmental justice. Am. J. Public Health.

[18] Dasgupta S., Bowen V.B., Leidner A. (2020). Association Between Social Vulnerability and a County’s Risk for Becoming a COVID-19 Hotspot—United States, June 1–July 25, 2020. MMWR Morb. Mortal Wkly Rep..

[19] SafeGraph (2020). Guide to Points-of-Interest Data: POI Data FAQ. SafeGraph. https://www.SafeGraph.com/points-of-interest-poi-data-guide.

[20] Ong L.L. (1997). Burgernomics: The economics of the Big Mac standard. J. Int. Money Financ..

[21] SafeGraph (2020). Monthly Places Patterns (aka “Patterns”) Jan 2018—Apr 2020. SafeGraph. https://docs.SafeGraph.com/docs/monthly-patterns.

[22] SafeGraph (2021). Personal correspondence.

[23] McKinney W. Data Structures for Statistical Computing in Python. Proceedings of the 9th Python in Science Conference.

[24] Reback J., McKinney W., Brockmendel J. (2020). pandas.

[25] Esri (2020). 2020 USA Median Household Income [basemap]. NYCOpenData. https://nyuds.maps.arcgis.com/home/item.html?id=20a60423d37c49ba9253526859ba93e1.

[26] Goldstein J., McKinley J. (2020). Coronavirus in N.Y.: Manhattan Woman Is First Confirmed Case in State. New York Times. https://www.nytimes.com/2020/03/01/nyregion/new-york-coronvirus-confirmed.html.

[27] Laefer D.F., Kirchner T., Cheong D. (2020). Data Resource Profile: Egress Behavior from Select NYC COVID-19 Exposed Health Facilities March–May 2020. arXiv.

[28] Waldman A. (2020). Life on a Block with an Emergency Morgue Truck: “We Hear the Hum of the Refrigerator Going All Night Long.” ProPublica. https://www.propublica.org/article/life-on-a-block-with-an-emergency-morgue-truck-we-hear-the-hum-of-the-refrigerator-going-all-night-long.

[29] Vigdor N. (2020). Several East Coast Universities Cancel Classes in Coronavirus Response. New York Times. https://www.nytimes.com/2020/03/08/nyregion/columbia-classes-canceled-coronavirus.html.

[30] NYC Office of the Mayor (2020). New York City to Close All School Buildings and Transition to Remote Learning. The Official Website of the City of New York. https://www1.nyc.gov/office-of-the-mayor/news/151-20/new-york-city-close-all-school-buildings-transition-remote-learning.

[31] Roy A., Kar B. Characterizing the Spread of COVID-19 from Human Mobility Patterns and Socio-demographic Indicators. Proceedings of the 3rd ACM SIGSPATIAL International Workshop on Advances on Resilient and Intelligent Cities.

[32] Lamb M., Kandula S., Shaman J. (2020). Differential COVID-19 case positivity in New York City neighborhoods: Socioeconomic factors and mobility. Influenza Other Respir. Viruses.

[33] Chang S., Pierson E., Koh P.W. (2021). Mobility network models of COVID-19 explain inequities and inform reopening. Nature.

[34] Azap R.A., Paredes A.Z., Diaz A., Hyer J.M., Pawlik T.M. (2020). The association of neighborhood social vulnerability with surgical textbook outcomes among patients undergoing hepatopancreatic surgery. Surgery.

[35] Segal A. (2010). Food Deserts: A Global Crisis in New York City Causes, Impacts and Solutions. Consilience.

[36] NYC Department of Buildings (2020). 2014 Construction Codes—Building Code. NYC Department of Buildings. https://www1.nyc.gov/site/buildings/codes/2014-construction-codes.page#bldgs.

